# Exploring the Dimensionality of the Perceived Cost of Learning High School Mathematics

**DOI:** 10.3390/ejihpe15120240

**Published:** 2025-11-26

**Authors:** Saule Raiziene, Lauryna Rakickiene, Dovile Butkiene

**Affiliations:** Institute of Psychology, Faculty of Philosophy, Vilnius University, Universiteto 9, LT-01513 Vilnius, Lithuania; lauryna.rakickiene@fsf.vu.lt (L.R.); dovile.butkiene@fsf.vu.lt (D.B.)

**Keywords:** expectancy–value theory, cost dimensionality, academic motivation, mathematics

## Abstract

The concept of cost, defined as the perceived negative consequences of engaging in a task, is an important yet understudied component of the expectancy–value theory of student motivation. In this study, we examined the multidimensional cost structure in mathematics learning, focusing on four facets proposed in recent research: effort, opportunity, emotional, and ego costs. Participants consisted of 1483 ninth-grade students from 24 public schools in Lithuania (56.04% girls; M = 14.88 years). Students completed a questionnaire developed to assess the four cost dimensions, along with measures of their intentions to pursue mathematics, procrastination, and negative classroom emotions; academic achievement data were obtained from school records. Using a split-sample design, we used exploratory and confirmatory factor analyses to test alternative structural representations of cost. A comparison of correlated-factor, higher-order, and bifactor models indicated that the correlated-factor ESEM model best fitted the data, supporting the specificity of the four distinct cost dimensions. Specific associations of each cost dimension with study outcomes further supported this differentiation, with ego cost most notably diverging from the other three cost dimensions. The results confirm the distinct nature of the four cost dimensions and underscore the importance of examining their separate roles in students’ mathematics learning, contributing to the existing evidence from a comparatively understudied cultural context.

## 1. Introduction

Understanding what drives students to engage in or withdraw from academic tasks is a central concern for educators, educational psychologists, and policymakers. One foundational framework for understanding motivation in achievement contexts is provided by the expectancy–value theory (EVT), developed by Eccles and colleagues (Eccles et al., 1983; Wigfield & Eccles, 2000), which has been recently renamed the situated expectancy–value theory, emphasizing the developmental, social, and cultural contexts in which these motivational processes unfold. Central to this theory is the premise that an individual’s choice, persistence, and performance in academic activities are influenced by two categories of beliefs: (1) their expectancies for success—how well students believe they will do on a task—and (2) the subjective value they attach to that task.

Within this framework, subjective task value has been elaborated to include four distinct components: intrinsic value, utility value, attainment value, and cost. Among these, cost refers to the perceived negative consequences of engaging in a task (Eccles et al., 1983; Eccles & Wigfield, 2020), capturing perceptions that may inhibit motivation, and is particularly relevant in academic domains such as mathematics, which is often associated with performance pressure, high-effort demands, and negative emotions (OECD, 2013). Gaspard et al. (2017) showed that mathematics, among other subjects, is marked by strong perceptions of cost; moreover, these perceptions increase with the grade level, indicating that adolescents perceive math as more costly than younger students. Given the persistent underrepresentation of students in STEM careers, it is especially important to understand how adolescents experience the cost of studying mathematics during the period in which they are making decisions that may shape their future career paths.

Despite being introduced four decades ago, the concept of perceived cost has received heightened attention only in recent years (e.g., Flake et al., 2015; Jiang et al., 2020; Kosovich et al., 2015; Lee et al., 2022; Luttrell et al., 2010; Muenks et al., 2023). Scholars such as Barron and Hulleman (2015) have even argued for the redefinition of the framework as the expectancy–value–cost model to underscore the distinct and critical role that perceived cost plays in shaping academic behavior. Recent empirical findings demonstrate that cost can negatively impact student engagement and performance independently of expectancy and value (Kim et al., 2022; Perez et al., 2019). Furthermore, cost has emerged as a key factor in profiles of the motivational beliefs of students, frequently differentiating between more and less adaptive patterns of motivation (Conley, 2012; Jiang & Zhang, 2023). However, despite the growing recognition of the importance of cost, there remains a lack of consensus regarding its definition and measurement. Building on recent conceptual and empirical developments, in this study, we examined how high school students perceive the cost of learning mathematics—specifically in terms of its potential dimensions and their interrelations.

### 1.1. Conceptualization and Differentiation of Cost Dimensions

Originally, cost was conceptualized as a multidimensional construct within the EVT framework, with the acknowledgement that different types of perceived barriers can undermine task engagement. In their early work, Eccles and colleagues (Eccles et al., 1983; Eccles, 2005; Wigfield & Eccles, 2000) proposed three theoretically distinct cost dimensions: the effort required to perform a task well (effort cost), the loss of opportunities for alternative valued activities (opportunity cost), and the psychological cost of failure (psychological/emotional cost). Despite this theoretical elaboration, the early empirical work did not differentiate among these dimensions. While some EVT instruments included items that touched on different aspects of cost, the measures were often not refined enough to capture these dimensions empirically. In many cases, items representing multiple cost types were combined into a single, undifferentiated scale (e.g., Battle & Wigfield, 2003; Chiang et al., 2011; Luttrell et al., 2010) or were merged into a composite value–cost score alongside positive value components (e.g., Buehl & Alexander, 2005). This practice precluded researchers from examining the possible distinct contributions and functional roles of the cost components proposed by Eccles and colleagues.

It was not until a decade ago that empirical evidence for the multidimensional nature of cost emerged, when Perez et al. (2014) confirmed three distinct cost factors through exploratory factor analysis. Using a cost measure adapted from Battle and Wigfield (2003), the authors differentiated three cost facets corresponding to those initially postulated by Eccles et al. (1983): effort cost, opportunity cost, and psychological cost—the latter operationalized to include both potential threats to self-worth in cases of failure and the anticipated negative emotions associated with engaging in the task. Subsequent studies built on this work by differentiating three cost dimensions, though with some variation in the definition of psychological/emotional cost. Some studies (e.g., Greene et al., 2023; Part et al., 2020; Perez et al., 2019) operationalized psychological cost in line with Perez et al. (2014), whereas others focused specifically on the anticipated negative emotions associated with engaging in a task, referring to this component as emotional cost (e.g., Fadda et al., 2020; Gaspard et al., 2015). Finally, Jiang (2015) and Jiang et al. (2020) proposed the four-dimensional cost operationalization, differentiating both the aspect of negative emotions (emotional cost) and threat to self-worth (ego cost). By using the label *ego cost* for this latter dimension, these authors avoided the confusion associated with the term *psychological cost*, which broadly encompasses both emotional and self-worth-related aspects.

In the present study, we follow the recently elaborated four-dimensional conceptualization of cost proposed by Jiang (2015) and Jiang et al. (2020), as the clear distinction between the emotional and ego-related aspects of cost not only addresses the long-standing conceptual ambiguity of psychological/emotional cost but is also supported by their distinct patterns of associations with other EVT variables and academic outcomes, as we elaborate later. Although there have been additional attempts to differentiate cost by introducing further facets—such as *outside cost*, which refers to the effort devoted to other tasks that leaves fewer resources for the focal task (Flake et al., 2015)—we did not include outside cost in the present study, as it was identified primarily in research with college students and reflects a level of contextual task evaluation that may not be developmentally appropriate for adolescents.

In line with the framework of Jiang (2015) and Jiang et al. (2020), we focused on the following four cost facets:

(1) **Effort cost**: Effort cost is defined as the excessively high, not worthwhile, and therefore aversive anticipated investment of effort and energy required to complete a task.
(2) **Opportunity cost**: Opportunity cost involves the perception that pursuing an academic task necessitates sacrificing other valued activities, highlighting the competing demands on adolescents’ time.
(3) **Emotional cost**: Emotional cost captures anticipated negative feelings, such as anxiety, stress, or annoyance, associated with engagement in a task.
(4) **Ego cost**: Ego cost refers to the threat that potential failure in a task poses to one’s self-concept or self-worth.

### 1.2. Modeling the Multidimensional Structure of Cost

While recent studies have demonstrated that cost can be empirically differentiated into distinct dimensions, a deeper conceptual question remains: do these dimensions truly reflect separate motivational constructs? In other words, is cost best understood as a set of independent experiences or as a unified perception with domain-specific manifestations? Addressing this question is essential for clarifying the structure of cost and for informing how it should be conceptualized and measured in educational research.

In this context, structural modeling has served as a tool for testing competing theoretical assumptions. Comparing first-order correlated-factor models to higher-order factor models provides insight into whether cost is best understood as a set of distinct yet related perceptions or as an overarching construct with multiple manifestations. In addition, bifactor modeling has recently been employed by researchers to further examine the functional independence versus interdependence of the cost dimensions by simultaneously estimating a general cost factor—capturing the variance common to all items—and specific factors representing distinct cost dimensions, such as the effort, opportunity, emotional, and ego cost dimensions (Jiang et al., 2020). These theoretical models can be tested within either a confirmatory factor analysis (CFA) framework or an exploratory structural equation modeling (ESEM) framework (Greene et al., 2023; Part et al., 2020). ESEM offers greater flexibility than CFA by allowing for incidental cross-loadings, which capture shared variance due to item wording or conceptual overlap and may therefore yield more accurate factor estimates (Alamer, 2022).

Over the past decade, some studies have examined the cost structure in isolation (Flake et al., 2015; Jiang et al., 2020; Perez et al., 2019), while others have modeled cost alongside positive value dimensions (e.g., Fadda et al., 2020; Gaspard et al., 2015; Gaspard et al., 2020; Greene et al., 2023; Jiang et al., 2018; Muenks et al., 2023; Part et al., 2020). Despite notable variability in the findings, several consistent patterns have emerged. First-order models with separate factors for each theoretical cost dimension tend to show a better fit than second-order models incorporating a higher-order general cost factor (Gaspard et al., 2015; Gaspard et al., 2020; Part et al., 2020). Furthermore, ESEM has generally outperformed CFA in modeling cost structures (Fadda et al., 2020; Greene et al., 2023; Part et al., 2020), likely because it can accommodate cross-loadings that reflect the conceptual overlap among cost types.

Additionally, CFA-based studies frequently report moderate to strong correlations among specific cost factors (Flake et al., 2015; Jiang et al., 2020; Perez et al., 2019), lending support to the theoretical plausibility of a general cost factor and the application of bifactor modeling. The bifactor structure of cost has received partial empirical support. While Fadda et al. (2020) concluded that a bifactor CFA model was not suitable, Jiang et al. (2020) found an acceptable fit. However, most of the reliable variance in the scale was attributed to a general cost factor, limiting the interpretive value of the specific cost dimensions. Only ego cost retained a meaningful degree of unique variance, suggesting it may represent a conceptually distinct aspect of cost not fully explained by the general factor. Part et al. (2020) and Greene et al. (2023), who modeled cost alongside value using ESEM, identified bifactor ESEM models as the best-fitting solution, though some concerns remained. For instance, some items from the psychological cost dimension did not load on the general factor in Part et al. (2020). Taken together, these findings highlight the complexity of the cost construct and suggest that while a general cost factor is often supported, specific dimensions—particularly ego cost—retain unique conceptual and empirical relevance.

### 1.3. Cost as Academic Outcome Predictor

As studies increasingly demonstrate that different cost dimensions can be meaningfully distinguished, a logical next step is to investigate whether these dimensions are differentially associated with important academic outcomes. However, the current evidence remains limited, as most studies have relied on composite cost scores to examine such relations. Much of this work has focused on associations between cost and academic achievement, consistently showing that higher cost is related to lower achievement scores (e.g., Jiang et al., 2018; Jiang & Rosenzweig, 2021; Trautwein et al., 2012). Importantly, Jiang et al. (2018) proved that composite cost predicted mathematics achievement even after accounting for self-efficacy and value. Other studies have linked general cost to a range of other academic outcomes, revealing negative associations with positive outcomes (e.g., persistence, future intention to pursue studying) and positive associations with maladaptive outcomes (e.g., procrastination, avoidance intentions, drop-out intentions, negative classroom affect) (Battle & Wigfield, 2003; Jiang et al., 2018; Jiang & Rosenzweig, 2021; Perez et al., 2014).

Within this generally consistent pattern of associations, an unexpected finding is the positive relationship between general cost and performance approach goals, noted by Jiang et al. (2018), which the authors suggest could be attributable to the inclusion of ego cost in the composite score—students who perceive high ego costs may adopt stronger performance approach goals in an effort to protect their self-worth. This interpretation underscores the value of examining separate cost dimensions to clarify their distinct associations with academic outcomes. In recent studies adopting this approach, researchers suggest that not all dimensions share the same pattern of relations. For example, Perez et al. (2019) used SEM and found that effort cost predicted achievement negatively, opportunity cost predicted it positively, and psychological cost showed no significant association. Opportunity cost also emerged as a positive predictor of achievement in a study by Greene et al. (2023), while neither effort nor psychological cost was significant. Similarly, Part et al. (2020) used a bifactor model that takes into account general value alongside specific value and cost dimensions and reported that opportunity cost positively predicted achievement. Greene et al. (2023) also found positive correlations between psychological cost and both performance avoidance and performance approach goals—a pattern consistent with Jiang et al.’s (2018) speculation that ego-related concerns embedded within cost may drive students to adopt stronger performance approach goals to protect their self-worth.

Further insight into the distinct roles of emotional and ego costs comes from recent work by Jiang et al. (2020) and Jiang and Zhang (2023), who provided correlations between all four cost dimensions and various academic outcomes. The authors showed that all four dimensions were positively associated with maladaptive academic behaviors, such as procrastination, disengagement, help-seeking avoidance, and test anxiety; however, more nuanced patterns also emerged. For instance, Jiang et al. (2020) found that only effort and emotional costs—and not opportunity or ego costs—were negatively related to achievement and future intentions. Opportunity cost was positively related to persistence, while effort and ego costs were not, and emotional cost was negatively related to persistence. Finally, although all four cost dimensions were positively associated with performance avoidance goals, ego and opportunity costs also showed positive associations with performance approach goals.

Taken together, these findings suggest that the distinct cost dimensions may exert different psychological pressures and influence educational outcomes through separate mechanisms. However, the current evidence base is still limited and somewhat inconsistent. In particular, results linking ego cost to performance approach goals—and its weaker or nonsignificant associations with other outcomes—hint that it may capture a unique aspect of the motivational experiences of students not fully explained by other cost dimensions. More research is needed to systematically disentangle the unique roles of each cost dimension in shaping the academic outcomes of students.

### 1.4. The Present Study

Despite the growing interest in the role of cost in expectancy–value theory, empirical studies continue to lag in both conceptual precision and methodological sophistication. As Eccles and Wigfield (2020) noted, we still lack a clear understanding of the cost structure. To this day, cost is often treated as a unitary construct despite the growing empirical evidence supporting its dimensionality, and even when its dimensional structure is acknowledged, there remains inconsistency regarding the number and nature of the distinct cost components. Furthermore, it is still an open question as to whether the cost dimensions are best understood as distinct experiences or as expressions of a general, overarching cost perception.

To date, the most comprehensive modeling effort that included cost was conducted by Part et al. (2020), who examined competing structural representations of subjective task value using first-order, higher-order, and bifactor models within both the CFA and ESEM frameworks. However, the study included only three cost components—effort, opportunity, and psychological costs—which prevented the differentiation of conceptually distinct aspects of emotional and ego costs. Moreover, in Part et al. (2020), cost was examined alongside other value facets, an approach well suited to clarifying how cost relates to other motivational beliefs within the broader framework of expectancy–value theory. However, given that the conceptualization of cost is still being refined (Eccles & Wigfield, 2024), modeling its dimensions in isolation, as was performed in our study, can serve for its subsequent integration into the broader framework of motivational beliefs outlined in expectancy–value theory.

Jiang et al. (2018) advanced cost research by differentiating emotional and ego costs, but their cost structure modeling was not as thorough and systematic as that of Part et al. (2020), and their research was also conducted in East Asian contexts. In one attempt at the cross-cultural validation of the four cost dimensions in South Korea, China, and Germany, the scales showed only weak measurement invariance across the countries. Moreover, ego cost demonstrated markedly different patterns of association with values—correlating positively in South Korea, negatively in China, and showing no correlation in Germany. Given that cultural norms around self-worth and academic failure may heighten the sensitivity to costs among East Asian students (Jiang & Zhang, 2023), it remains uncertain how meaningful these four cost dimensions are in other cultures.

In this study, we examine the multidimensional structure of cost, drawing on the framework proposed by Jiang (2015) and Jiang et al. (2020). To achieve this aim, we pose two research questions: (1) Is the four-dimensional structure of cost (effort, opportunity, emotional, and ego costs) evident in a sample of Lithuanian adolescents when evaluated using advanced modeling approaches? (2) Is the differentiation of the cost dimensions supported by their specific relations to academic outcomes, i.e., achievement, intention to pursue mathematics in the future, procrastination, and negative classroom affect.

This investigation is situated in the Lithuanian educational context. In Lithuania, the ninth grade marks the beginning of upper secondary education and is the first year of high school. At this stage, most students transition to new schools, making it a period of academic and social adjustment. Mathematics holds a particularly high-stakes position within the Lithuanian education system: a passing score on the mathematics examination is required for qualifying for university or college admission. Consequently, mathematics is often perceived as a high-pressure subject by Lithuanian adolescents.

## 2. Materials and Methods

### 2.1. Participants and Procedure

The data came from the longitudinal research project “Towards the Effective Learning: Analysis of the Psychological Mechanisms of Obstacles to Learning Mathematics”, which explored the motivational beliefs and learning experiences in mathematics from the beginning of the ninth grade throughout two years up to the end of basic education. The project sample comprised 1649 students (69% recruitment rate). Project data were collected from 89 classes in 24 public schools throughout Lithuania, using quota sampling to ensure coverage of urban, town, and rural contexts. In the current study, we used data from the first phase of the project.

A total of 1483 ninth graders participated in this study, consisting of 644 boys (43.44%) and 831 girls (56.04%). Students were 14.88 years old on average (*SD* = 0.34), and a total of 620 participants (41.81%) attended schools in cities, 631 (42.55%) in towns, and 232 (15.64%) in rural areas, approximating the national distribution of students in higher grades according to the location of the school attended.

The data were collected in late autumn 2023 (approximately 9–13 weeks after the start of the school year). Only students for whom written informed parental consent had been obtained were invited to participate in this study. Participation was voluntary; students provided their own assent before completing the questionnaires and were informed that they could withdraw at any time without consequences. The questionnaires were administered by trained research assistants during regular classroom hours. All data were anonymized and stored separately from any personally identifying information. The procedures for the data collection and storage were approved by the Committee on Research Ethics of the Institute of Psychology at Vilnius University (protocol code 20/(1.13 E) 250000-KT-161, 2 October 2023).

### 2.2. Measures

All survey items were written in Lithuanian, focused on the subject of mathematics, and were rated on a 5-point Likert scale ranging from 1 = *completely disagree* to 5 = *completely agree* (for perceived cost and intention to pursue mathematics) and from 1 = *does not apply* to 5 = *fully applies* (for procrastination and negative emotions).

**Perceived cost.** In designing the cost measurement instrument, we drew on (1) the four-dimensional structure proposed by Jiang (2015) and Jiang et al. (2020), and (2) a review of the facets of these dimensions evident in items employed in previous EVT research. The final instrument consisted of 20 items (5 items per cost dimension), the majority adapted from prior studies (Flake et al., 2015; Gaspard et al., 2020; Jiang et al., 2020; Lee et al., 2022; Luttrell et al., 2010; Perez et al., 2019), complemented by self-developed items. A full list of the cost items in Lithuanian and English is provided in Appendix A.

The *effort cost* items reflect the excessive effort involved in studying mathematics and the energy that it demands. Although some authors consider not only excessive effort but also the time invested in a task when measuring effort cost, we chose not to include items related to time investment to avoid overlap between the effort cost and opportunity cost items that could arise from similar wording (i.e., repeated references to “time”). The *opportunity cost* items emphasize the sacrifices students make when studying mathematics, including giving up other valuable activities, leisure time, and time with peers. The *emotional cost* items reflect anticipated emotions related to the subject of math and the process of learning it, encompassing both negative emotions in general and specific emotions such as stress, anxiety, and annoyance. The *ego cost* items refer to disappointment in oneself, feelings of embarrassment, and fear of being seen as lacking ability by others in the event of failing math. Although Jiang et al. (2020) conceptualized ego cost primarily as a threat to self-worth arising from the fear of being negatively evaluated by others, we also included items reflecting threats to self-worth arising from internal self-evaluation. This adaptation reflects cultural differences, as Jiang’s work was conducted in a collectivistic context, whereas our study is situated in a more individualistic one. Similarly, Perez et al. (2019) and Lee et al. (2022), who conducted their research in Western contexts, included items capturing both aspects when measuring psychological cost.

**Academic outcomes.** We measured both positive (achievement in mathematics, intention to pursue mathematics) and negative (procrastination, negative class emotions) academic outcomes when examining the relationship between the cost dimensions and learning behavior.

The *intention to pursue mathematics* was measured via three items used in the study by Chung and Kim (2022). The items reflect how strongly an individual is considering a career involving mathematics (e.g., “I intend to enter a career that will use math”). The internal consistency of the measure was excellent (Cronbach α = 0.91).

*Procrastination* was measured via five items adapted from Jiang et al. (2018). The items assess procrastination in studying math (e.g., “I delay studying math until it is too late”). The internal consistency of the measure was adequate (Cronbach α = 0.77).

*Negative emotions* experienced during math class were measured by five self-developed items. The items refer to feelings such as anxiety, frustration, and disappointment (e.g., “I feel hopeless during math class”). The internal consistency of the measure was good (Cronbach α = 0.88).

*The achievement in the mathematics index* was based on the final grade from the first semester (on a 10-point scale), obtained from official school records.

### 2.3. Data Analysis

The purpose of this study was to gather evidence about the cost multidimensionality. In the structural phase of the data analysis, we identified and confirmed the underlying cost dimensionality of the set of items selected to measure different aspects of cost, following the recommendations made by Worthington and Whittaker (2006). The total sample (N = 1483) was randomly divided into two subsamples: Subsample A (N = 463; 30%), which was used for exploratory factor analysis (EFA) to identify the underlying factor structure, and Subsample B (N = 1020; 70%), which was used for confirmatory factor analysis (CFA) to replicate and validate the identified cost structure. This unequal split was intentional, as CFA generally requires larger sample sizes to ensure model stability and adequate statistical power (Brown, 2015). In the external phase, the predictive utility of the cost dimensions was expressed through its associations with different academic outcomes.

The EFA and CFA were conducted in Mplus 8.9. The Maximum Likelihood Robust (MLR) estimator was applied. To handle missing data, Full Information Maximum Likelihood (FIML) was used, as the data in both subsamples were probably missing at random based on normed Little’s MCAR test (i.e., χ^2^/df = 1.17 for Subsample A; 1.16 for Subsample B), indicating a good fit between the sample scores with and without imputations (Bollen, 1989). The Type = Complex option was used to account for the nested structure of the data (students nested within classes), with classroom membership as the clustering variable, correcting standard errors and chi-square tests of the model fit (Muthén & Muthén, 2017). In line with Rhemtulla et al.’s (2012) guidance, the indicators were treated as continuous given the five-category response format and large sample size.

The overall model fit was evaluated using the chi-square (χ^2^) value and the degrees of freedom, as well as the comparative fit index (CFI), Tucker–Lewis index (TLI), and root-mean-square error of approximation (RMSEA). According to commonly accepted cut-off criteria (Hu & Bentler, 1999), CFI and TLI values greater than 0.95 and RMSEA values below 0.06 indicate a good model fit with the data.

To determine the number of cost dimensions, the EFA was conducted on 20 cost items representing four theorized dimensions: effort cost, opportunity cost, emotional cost, and ego cost, using Subsample A. Oblimin rotation was applied, as we expected the factors to be correlated. Models with one to five factors were estimated. The decision regarding the optimal factor structure was informed by parallel analysis (O’Connor, 2000), conducted using the “psych” package in R (Revelle, 2020). The item retention was determined according to Worthington and Whittaker’s (2006) guidelines, requiring substantial primary loadings (≥0.40) on the target factor and minimal cross-loadings (<0.32) on the non-target factors to ensure factor purity.

Subsequently, we conducted the CFA using Subsample B to validate the cost dimension structure identified during the EFA. To confirm the best-fitting cost dimension structure, we evaluated three general theoretical models—a correlated-factor (CF) model, higher-order (H) model, and bifactor (B) model—each tested using both the CFA and ESEM frameworks.

In the correlated-factor models, the cost items were specified for loading onto their designated cost dimension factor, with factor correlations freely estimated. In the higher-order models, the cost items were loaded onto first-order dimension factors, which, in turn, were loaded onto a higher-order cost factor. In the bifactor models, all items were loaded onto both a general cost factor and one specific cost factor.

The CF-, H, and B-ESEM models were specified as structural analogues to their CFA counterparts in terms of the target item loadings and factor correlations; however, cross-loadings were freely estimated in the ESEM. The ESEM code generator for Mplus developed by De Beer and Van Zyl (2019) was used to generate the syntaxes for the ESEM models. We evaluated each model based on the overall fit to the data, pattern of factor loadings, and correlations between factors. To determine the most appropriate dimensional structure of the cost construct, we further compared the models by examining the changes in the CFI (ΔCFI) and RMSEA (ΔRMSEA). A substantially better model fit is indicated when the change in the CFI exceeds 0.010 and the change in the RMSEA exceeds 0.015 (Chen, 2007; Hu & Bentler, 1999).

Following the recommendations of Morin et al. (2020), McDonald’s omega coefficients of composite reliability (McDonald, 1970, 1999) were calculated using the standardized item loadings and uniqueness from the retained measurement model representing the cost dimensions.

In the final (external) phase of the analysis, we examined associations between the cost dimensions—based on the retained measurement model—and academic outcomes, including math achievement, intention to pursue mathematics, procrastination, and negative emotions.

**Declaration of generative AI and AI-assisted technologies in the writing process:** During the preparation of this work, the authors made use of *ChatGPT* (*OpenAI*, *GPT-5*) to assist in the language polishing, specifically to enhance the readability and flow. The authors carefully reviewed and revised all content generated with the assistance of this tool and accept full responsibility for the final content of the published article.

## 3. Results

### 3.1. Structural Phase: Exploratory Factor Analysis

The EFA was conducted to uncover the potential dimensionality of the cost construct. The outcomes of the EFA from one to five factors are shown in Table 1.

The EFA results indicated that the one- and two-factor models did not fit the data, while the results of the three-factor model showed an improved fit but remained suboptimal. In contrast, both the four- and five-factor solutions demonstrated good model fit, suggesting that these solutions were more appropriate for the data. The results of the parallel analysis supported the four-factor model, which also demonstrated the most conceptually coherent factor-loading pattern. In comparison, the fifth factor in the five-factor solution did not display a clear or conceptually meaningful loading pattern. The items loaded on this factor were few and inconsistent, suggesting that it may reflect a residual or method-related component rather than a meaningful construct (Brown, 2015). Accordingly, the final retained four-factor model comprised five items per factor.

The standardized factor loadings of the four-factor model are reported in Table 2. According to the item contents, the four factors represented effort, opportunity, emotional, and ego cost. All items had greater than 0.40 primary loadings on their hypothesized factor, with the exception of two items (i.e., eff1 and eff4). Although eff1 and eff4 were initially expected to load on the Effort Cost factor, exploratory results showed their primary loadings on the Emotional Cost factor, which may be explained by their phrasing in Lithuanian (see Appendix A), which may have been interpreted by respondents as indicating the depletion of emotional resources rather than perceived effort. As such interpretations do not align with the conceptual understanding of effort cost and emotional cost (i.e., anticipation of negative emotions), these items were excluded from the subsequent analyses. Furthermore, with the exception of two items (opp4 and emo4) that cross-loaded above 0.32 on a non-target factor, all other items loaded on their intended single factors. Thus, the EFA provided initial support for the hypothesized four-factor cost structure.

### 3.2. Structural Phase: Confirmatory Factor Analysis

Based on the EFA results suggesting a four-factor model, we conducted a series of CFAs to test and validate the proposed four-dimensional cost structure. Specifically, we evaluated the fit of two correlated-factor models (Models 1 and 2), two higher-order models (Models 3 and 4), and two bifactor models (Models 5 and 6). Table 3 presents a summary of the model fit indices for each of the six estimated models.

**Correlated-factor models:** According to the results, of the two correlated-factor models tested, the CF-ESEM model (Model 2) demonstrated a better fit with the data across all fit indices. In this model (see Table 4), all four factors were clearly defined by their target loadings: effort cost (|λ| = 0.494–0.925, M = 0.684); opportunity cost (|λ| = 0.595–0.913, M = 0.776); emotional cost (|λ| = 0.561–0.825, M = 0.726); and ego cost (|λ| = 0.512–0.902, M = 0.741). The non-target loadings were consistently smaller than the target loadings: |λ| = 0.003–0.246 (M = 0.027) for effort cost; |λ| = 0.007–0.223 (M = 0.036) for opportunity cost; |λ| = 0.000–0.240 (M = 0.030) for emotional cost; and |λ| = 0.002–0.130 (M = 0.009) for ego cost. There were a sufficient number of non-trivial cross-loadings (|λ| > 0.10), suggesting the presence of construct-relevant multidimensionality due to item fallibility. Correlations between the cost dimensions (Table 5) ranged from 0.089 to 0.695 (M = 0.409), with ego cost showing the lowest correlations with the other facets (|r| = 0.089–0.196, M = 0.136).

**Higher-order models:** Similar to the correlated factor models, which served as a basis for constructing the higher-order models, the H-CFA model (Model 3) did not have a good fit with the data, whereas the H-ESEM model (Model 4) did. The comparison between the CF-ESEM (Model 2) and H-ESEM (Model 4) models did not indicate a meaningful difference in the overall fit. However, in the H-ESEM model, the second-order factor explained substantial proportions of the variance in the effort cost, opportunity cost, and emotional cost first-order factors (62%, 75%, and 67%, respectively) but only 2.6% in the ego cost factor, revealing a notable limitation of the higher-order cost construct in capturing this dimension.

**Bifactor models:** Both Models 5 and 6 demonstrated a good fit with the data; similarly, as with the correlated-factor and higher-order models, the B-ESEM solution (Model 6) showed a better fit. Neither the B-ESEM (Model 6) nor the CF-ESEM (Model 2) showed a clear advantage over the other based on a comparison of the model fit indices. According to an analysis of the factor–item associations in the B-ESEM model (Model 6) (see Supplementary Materials, Table S1), the general cost factor was well defined by most indicators (|λ| = 0.013–0.802, M = 0.543). However, two items measuring ego cost had trivial loadings on this general factor. Moreover, the indicators of ego cost (|λ| = 0.013–0.375, M = 0.135) showed weaker associations with the cost G-factor than those of effort cost (|λ| = 0.783–0.802, M = 0.790), opportunity cost (|λ| = 0.524–0.783, M = 0.701), and emotional cost (|λ| = 0.496–0.722, M = 0.665). Beyond the G-factor, the specific factors for effort cost (|λ| = 0.101–0.456, M = 0.325) and emotional cost (|λ| = 0.226–0.486, M = 0.401) were not well defined, as none of the indicators loaded on these specific factors above 0.500, and only one of the four loadings exceeded 0.500 for the opportunity cost S-factor (|λ| = 0.255–0.527, M = 0.417). In contrast, the ego cost S-factor was well defined (|λ| = 0.529–0.890, M = 0.735). Taken together, these results indicate that effort, opportunity, and emotional cost facets are largely captured by the general factor, while ego cost is more distinctly represented at the specific-factor level. Thus, despite the acceptable fit of the B-ESEM model, the lack of well-defined specific effort, opportunity, and emotional cost facets suggests that the bifactor model does not adequately capture the cost structure.

Thus, the CF-ESEM model (Model 2) appeared to provide the most plausible representation of the cost construct’s dimensional structure. All four scales of this model—effort cost, opportunity cost, emotional cost, and ego cost—showed good reliability, with McDonald’s omega values of 0.821, 0.906, 0.873, and 0.871, respectively.

### 3.3. External Phase: Correlational Analysis

Drawing on the measurement structure of the cost construct established through structural analyses, the external phase provided additional converging evidence for the relevance of the cost dimensions, based on their associations with the academic outcomes of students. The results of the correlational analysis conducted on Subsample B (N = 1020) are presented in Table 5.

The correlation analysis revealed a consistent pattern of associations between the effort, opportunity, and emotional cost dimensions and academic outcomes, whereas ego cost demonstrated a distinct and weaker pattern of relationships. Specifically, effort, opportunity, and emotional costs were negatively correlated with mathematics achievement and the intention to pursue mathematics and were positively associated with procrastination and negative emotions. Effort and opportunity costs showed highly similar correlation patterns across the academic outcomes, with the exception that effort cost was more strongly related to mathematics achievement. In contrast, emotional cost stood out by demonstrating stronger associations with all outcomes except achievement, as well as a particularly strong association with negative emotions.

Ego cost showed a weak positive association with academic outcomes, with the exception of procrastination, for which the association was not statistically significant. However, among these, only the association with negative emotions aligned in direction with the patterns observed for the other cost dimensions.

## 4. Discussion

In this study, using a large sample of high school students and focusing specifically on the dimensionality of the cost construct, we found consistent evidence across the subsamples, using both the EFA and CFA approaches, for the four distinct cost dimensions: effort, opportunity, emotional, and ego costs. These findings align with Eccles’ theoretical conceptualization of cost, which encompasses multiple facets—effort, opportunity, and psychological/emotional facets (Eccles et al., 1983; Eccles, 2005; Wigfield & Eccles, 2000)—with the latter further differentiated into emotional and ego costs (Jiang, 2015; Jiang et al., 2020).

Although the strong correlations among the cost dimensions may suggest the existence of a general cost factor (Flake et al., 2015; Gaspard et al., 2020; Perez et al., 2014), in our study, the CF-ESEM model (Model 2) emerged as the most appropriate representation of the data after systematically testing and comparing the correlated-factor, higher-order, and bifactor models. While the H-ESEM model (Model 4), in which the cost dimensions load onto a higher-order construct, and the B-ESEM model (Model 6), which accounts for both generality and specificity within the cost model, also provided good fit to the data, a closer inspection revealed important shortcomings. In the higher-order model, the general factor accounted for very little of the variance in the ego cost dimension, whereas in the bifactor model, the specific factors of effort, emotional, and opportunity costs were not well defined beyond the general factor. These results suggest that a single overarching cost factor does not adequately capture the unique features of effort, opportunity, emotional, and ego costs.

Studies have often supported a bifactor structure of task value when cost is examined alongside other values (e.g., Fadda et al., 2020; Part et al., 2020). However, even within these investigations, the evidence points to the distinctiveness of the specific cost dimensions rather than their integration into a general cost factor. This pattern highlights the specificity of the cost dimensions rather than the generality of cost, which is consistent with our findings.

The specificity of the cost dimensions is reflected not only in the investigation of the cost structure but also in the diversity of their associations with outcomes. Only three of the four cost dimensions displayed similar association patterns. We found that effort, opportunity, and emotional costs were negatively associated with mathematics achievement and the intention to pursue mathematics in the future but were positively associated with procrastination and negative classroom affect. Other researchers have likewise reported a general pattern in which the cost dimensions show negative associations with positive outcomes and positive associations with negative outcomes (Jiang et al., 2020; Jiang & Zhang, 2023).

At the same time, even among these three dimensions, certain differences emerged. Emotional cost stood out from effort and opportunity costs: effort and opportunity costs followed a similar pattern and had stronger negative associations with positive outcomes than emotional cost, whereas emotional cost was more strongly related to negative outcomes. These differential associations may be interpreted in light of Part et al.’s (2020) argument that, according to social exchange theory, the cost dimensions reflect different forms of commitment. Effort and opportunity costs, which were strongly tied to achievement and aspirations in our study, reflect behavioral commitments, as they directly involve time and energy investment and the sacrifice of valued alternative activities. Emotional cost, in contrast, appears to be more closely tied to affective commitments, as it reflects the anticipation of negative emotions experienced when engaging in a task, such as anxiety or frustration.

Furthermore, emotional cost correlated particularly strongly with negative classroom affect in our study. On the one hand, this might reflect the jangle fallacy: recent evidence by Song et al. (2023) suggests that emotional cost and negative classroom emotions may be difficult to disentangle when investigated through student self-report measures. On the other hand, the two constructs are conceptually distinct: whereas classroom emotions capture lived affective experiences in the moment, emotional cost refers to the anticipation of such experiences (Jiang et al., 2018, 2020). This prospective quality of emotional cost explains its particularly close ties to classroom affect and justifies its treatment as a distinct motivational belief. It is important to emphasize this prospective quality when constructing emotional cost measures, as some emotional experiences—anxiety in particular—may function as both anticipation and lived experience, thereby reinforcing the observed links between these constructs (Song et al., 2023).

In contrast, the correlations between ego cost and the other dimensions were weak in the retained model, and ego cost revealed a distinct pattern of associations. Unlike the other three dimensions, ego cost correlated positively—albeit modestly—with positive outcomes, namely, mathematics achievement and the intention to pursue mathematics, and showed no significant relation to procrastination. The only outcome it shared with the other cost dimensions was a positive association with negative classroom affect. This distinctive profile resonates with previous studies that found that ego-related concerns do not align neatly with the detrimental pattern more typically observed for the other cost dimensions (Greene et al., 2023; Jiang et al., 2020). Although this pattern could raise questions about whether ego cost is genuinely a cost—as its relation to positive study outcomes may render it value-like—EVT defines value as perceived benefits and cost as the perceived negative implications of engagement (Eccles et al., 1983; Eccles & Wigfield, 2020). Ego cost aligns with the latter, as it reflects a perceived threat.

These findings may be interpreted in light of the research of Jiang et al. (2018), who speculated that heightened ego concerns may push students to more strongly strive for achievement to protect their self-worth. This interpretation is consistent with prior findings on the positive associations between ego cost and performance approach goals (Jiang et al., 2020) and resonates with Part et al.’s (2020) argument that the cost dimensions reflect different forms of commitment. Although both ego and emotional costs appear to operate through negative emotions, their origins may differ. Ego cost may be linked more closely to the anticipation of failure and the threat it poses to self-worth, consistent with Eccles’ original definition of psychological cost (Eccles et al., 1983), whereas emotional cost may relate more to the anticipation of excessive, unrewarding effort, as highlighted by Flake (2012). These potentially distinct mechanisms could explain why ego cost shows a more complex pattern of associations compared to the generally more detrimental emotional cost profile.

Our results encourage a broader discussion about the quality of motivation. The process through which negatively valenced motivational forces promote student engagement is well documented in major theories of achievement motivation; for example, performance-avoidance goals in achievement goal theory (Elliot & McGregor, 2001) and introjected regulation in self-determination theory (Ryan & Deci, 2000) may lead students to study harder, yet they remain psychologically detrimental in terms of well-being. This has not been previously emphasized in EVT, where cost has traditionally been treated as uniformly negative and a cause of disengagement. Differentiating ego cost enables a more refined understanding by showing that the mechanisms that underlie the different cost facets can operate in distinct ways and do not necessarily result in uniformly negative consequences.

In addition to the theoretical implications, the present findings underscore the methodological benefits of employing an ESEM framework to examine the dimensionality of cost. Relative to a traditional CFA representation, the ESEM solution not only demonstrated a superior model fit but also produced less intercorrelated cost dimensions, thereby yielding a more differentiated and conceptually coherent representation of the construct. The retention of a CF-ESEM model further carries important implications for the computation of observed cost scores. Specifically, while CFA-based approaches typically disregard cross-loadings and thus risk overlooking construct-relevant multidimensionality, the ESEM framework explicitly accommodates such complexities arising from item fallibility (Morin et al., 2016). As a result, the scale scores derived from the ESEM model are corrected for measurement error (Morin et al., 2016), allowing them to retain the underlying nature of the latent constructs while offering a more accurate and refined operationalization of the cost dimensions (Alamer, 2022).

***Limitations and future directions:*** Despite its contributions, this study has several limitations that should be acknowledged. To begin with, we modeled the cost structure without considering other motivational beliefs postulated in EVT. This approach is justified by the fact that the conceptualization of cost is still being refined (Eccles & Wigfield, 2024), and examining its dimensions in isolation can provide a foundation for its subsequent integration into the broader framework of motivational beliefs. Nevertheless, because cost is theorized as a negative aspect of value within EVT (Eccles et al., 1983), the next step is to examine the four cost dimensions alongside other value components to ensure their distinctiveness.

The next limitation concerns the generalizability of the results. Although our study benefits from a relatively large sample that captures diversity among students in terms of school location (city, town, rural area), it is limited by being drawn from a single country (Lithuania) and age cohort (ninth-grade students). The Lithuanian educational system places considerable pressure on students to learn mathematics, and the ninth grade represents a transitional stage from lower to upper secondary education—conditions that may have contributed to shaping the perceptions of students regarding learning mathematics. Consequently, replication studies are needed to examine the generalizability of the four-dimensional cost structure, as identified in the first-order ESEM model, to other samples, including students of different age groups and from different cultural contexts. Moreover, in the present study, we focused exclusively on the mathematics domain, and future research should investigate whether the identified cost dimension structure extends to other academic domains.

Furthermore, our approach to revealing the specificity of the cost dimensions through their associations with academic outcomes has certain limitations. First, with the exception of mathematics achievement, both the cost dimensions and academic outcomes were assessed via self-report. Although self-report instruments are widely used for measuring these constructs, they are vulnerable to mono-method bias, which may inflate or attenuate observed associations. In addition, the associations were examined without controlling for prior academic achievement, which could differentially affect both the cost dimensions and academic outcomes. Finally, this study was correlational in nature, which precludes any causal inference regarding the relations between the cost dimensions and academic outcomes. Thus, future research on cost dimensionality would benefit from incorporating multiple data sources (e.g., behavioral indicators, teacher ratings), considering prior achievement, and using longitudinal designs to clarify the psychological mechanisms underlying the effects of the distinct cost dimensions, thereby strengthening the evidence for their specificity.

## 5. Conclusions

The results of this study highlight the importance of treating cost as a multidimensional construct and of differentiating between effort, opportunity, emotional, and ego cost facets, as each reflects qualitatively unique aspects of the learning experiences of students. Only a small number of studies have explicitly distinguished all four cost dimensions—including ego cost from emotional cost—across cultural settings (Gaspard et al., 2020; Jiang et al., 2018, 2020). By extending this work to Lithuania, we contribute evidence from an Eastern European context that has so far been largely absent from student motivation research. Our findings therefore provide further support for the meaningful differentiation of the four cost dimensions in the mathematics learning experiences of students across cultures, even when assessed with different measures.

## Figures and Tables

**Table 1:** Goodness-of-fit statistics for exploratory factor analysis (Subsample A, N = 463).

Model	Chi-Square (df)	CFI	TLI	RMSEA [90% CI]
One factor	1547.89 (170) ***	0.735	0.704	0.132 [0.126, 0.138]
Two factors	721.83 (151) ***	0.890	0.862	0.090 [0.084, 0.097]
Three factors	411.15 (133) ***	0.947	0.924	0.067 [0.060, 0.075]
Four factors	209.84 (116) ***	0.982	0.970	0.042 [0.033, 0.051]
Five factors	179.02 (100) ***	0.985	0.971	0.041 [0.031, 0.051]

*Note.* *** *p* < 0.001.

**Table 2:** Standardized factor loadings from four-factor EFA solution (N = 463).

Item	Effort Cost	Opportunity Cost	Emotional Cost	Ego Cost
	λ (SE)	λ (SE)	λ (SE)	λ (SE)
eff1 ^a^	−0.031 (0.104)	0.226 (0.090) *	0.477 (0.079) *	−0.019 (0.040)
eff2	0.770 (0.071) *	0.113 (0.073)	0.004 (0.036)	−0.022 (0.024)
eff3 ^a^	0.813 (0.055) *	−0.033 (0.035)	0.077 (0.049)	0.047 (0.028)
eff4	0.195 (0.077) *	0.282 (0.073) *	0.410 (0.068) *	−0.069 (0.035) *
eff5	0.740 (0.079) *	−0.002 (0.058)	0.119 (0.067)	−0.043 (0.032)
opp1	0.099 (0.071)	0.690 (0.057) *	−0.137 (0.052) *	0.104 (0.039) *
opp2	−0.065 (0.045)	0.793 (0.049) *	0.152 (0.045) *	0.013 (0.029)
opp3	−0.004 (0.044)	0.800 (0.061) *	0.118 (0.055) *	0.001 (0.028)
opp4 ^b^	0.446 (0.083) *	0.526 (0.079) *	−0.058 (0.034)	−0.030 (0.027)
opp5	0.050 (0.056)	0.824 (0.054) *	0.022 (0.041)	−0.053 (0.022) *
emo1	0.031 (0.072)	−0.040 (0.054)	0.751 (0.062) *	0.001 (0.029)
emo2	0.034 (0.064)	0.095 (0.059)	0.740 (0.068) *	0.007 (0.029)
emo3	0.162 (0.069) *	0.117 (0.051) *	0.618 (0.064) *	0.052 (0.032)
emo4 ^b^	0.342 (0.087) *	0.056 (0.063)	0.440 (0.079) *	0.071 (0.033) *
emo5	0.010 (0.055)	0.006 (0.042)	0.855 (0.043) *	0.003 (0.025)
ego1	0.010 (0.076)	0.108 (0.067)	−0.145 (0.062) *	0.600 (0.040) *
ego2	0.027 (0.057)	−0.040 (0.058)	−0.019 (0.049)	0.763 (0.026) *
ego3	0.104 (0.074)	0.029 (0.072)	0.182 (0.066) *	0.636 (0.043) *
ego4	−0.104 (0.058)	0.013 (0.046)	0.023 (0.045)	0.776 (0.028) *
ego5	0.004 (0.051)	−0.025 (0.043)	−0.013 (0.042)	0.862 (0.022) *

*Note.* * *p* < 0.05; λ—standardized factor loading; SE—standard error; ^a^ Item with poor loading on the intended factor; ^b^ Item with cross-loading greater than 0.32.

**Table 3:** Goodness-of-fit statistics for different confirmatory factor analysis models (Subsample B, N = 1020).

Model	Model Type	Chi-Square (df)	CFI	TLI	RMSEA [90% CI]	Compared With	ΔCFI	ΔRMSEA
M1: First-order CFA model (four correlated factors)	CF-CFA	684.98 (129) ***	0.940	0.928	0.065 [0.060, 0.070]			
M2: First-order ESEM model (four correlated factors)	CF-ESEM	238.35 (87) ***	0.984	0.971	0.041 [0.035, 0.048]	Model 1	0.044	−0.024
M3: Higher-order CFA model (one second-order and four first-order factors)	H-CFA	707.32 (131) ***	0.937	0.927	0.066 [0.061, 0.070]	Model 2	−0.047	0.025
M4: Higher-order ESEM model (one second-order and four first-order factors)	H-ESEM	239.83 (89) ***	0.984	0.972	0.041 [0.035, 0.047]	Model 2	0.000	0.000
M5: Bifactor model (one general and four specific factors)	B-CFA	423.73 (117) ***	0.967	0.956	0.051 [0.046, 0.056]	Model 2	−0.017	0
M6: ESEM bifactor model (one general and four specific factors)	B-ESEM	135.52 (73) ***	0.993	0.986	0.029 [0.021, 0.037]	Model 5 Model 2	0.026 0.009	−0.022 −0.012

*Note.* *** *p* < 0.001.

**Table 4:** Standardized factor loadings from CF-ESEM model (Subsample B, N = 1020).

Item	Effort Cost	Opportunity Cost	Emotional Cost	Ego Cost
	λ (SE)	λ (SE)	λ (SE)	λ (SE)
eff2	0.634 (0.037) ***	0.223 (0.035) ***	0.042 (0.029)	−0.034 (0.015) *
eff3	0.925 (0.038) ***	−0.123 (0.027) ***	0.048 (0.027)	0.051 (0.011) ***
eff5	0.494 (0.049) ***	0.214 (0.038) ***	0.168 (0.038) ***	−0.077 (0.024) **
opp1	0.173 (0.042) ***	0.625 (0.040) ***	−0.169 (0.039) ***	0.130 (0.026) ***
opp2	−0.115 (0.031) ***	0.857 (0.032) ***	0.113 (0.031) ***	0.016 (0.017)
opp3	−0.124 (0.024) ***	0.913 (0.029) ***	0.090 (0.023) ***	−0.044 (0.018) *
opp4	0.246 (0.035) ***	0.595 (0.042) ***	0.050 (0.035)	−0.002 (0.020)
opp5	0.040 (0.029)	0.891 (0.033) ***	−0.051 (0.026)	−0.011 (0.016)
emo1	−0.089 (0.042) *	−0.051 (0.034)	0.776 (0.042) ***	0.016 (0.025)
emo2	−0.013 (0.032)	0.011 (0.032)	0.825 (0.027) ***	0.010 (0.021)
emo3	0.110 (0.036) **	0.055 (0.035)	0.704 (0.038) ***	0.053 (0.180) **
emo4	0.217 (0.047) ***	0.019 (0.043)	0.561 (0.049) ***	0.008 (0.022)
emo5	0.003 (0.040)	0.079 (0.035) *	0.766 (0.050) ***	0.004 (0.023)
ego1	−0.080 (0.050)	0.027 (0.048)	0.015 (0.055)	0.512 (0.028) ***
ego2	−0.038 (0.033)	−0.010 (0.031)	0.000 (0.029)	0.817 (0.018) ***
ego3	0.096 (0.037)**	−0.027 (0.033)	0.240 (0.038) ***	0.680 (0.019) ***
ego4	−0.012 (0.033)	0.007 (0.031)	−0.084 (0.034) *	0.796 (0.020) ***
ego5	−0.013 (0.031)	0.043 (0.024)	−0.069 (0.028) *	0.902 (0.016) ***

*Note.* *** *p* < 0.001; ** *p* < 0.01; * *p* < 0.05; λ—standardized factor loading; SE—standard error.

**Table 5:** Correlations among four cost dimensions and students’ academic outcomes (Subsample B, N = 1020).

	Cost Dimensions			
	Effort Cost	Opportunity Cost	Emotional Cost	Ego Cost
*Latent correlations between four cost dimensions in CF-ESEM model*				
Effort cost	-	0.698 ***	0.655 ***	0.089 *
Opportunity cost		-	0.695 ***	0.122 **
Emotional cost			-	0.196 ***
*Correlations among four cost dimensions and academic outcomes*				
Achievement	−0.507 ***	−0.408 ***	−0.388 ***	0.183 ***
Intention to pursue mathematics	−0.298 ***	−0.272 ***	−0.339 ***	0.173 ***
Procrastination	0.264 ***	0.256 ***	0.385 ***	−0.058
Negative emotions	0.557 ***	0.603 ***	0.807 ***	0.293 ***

*Note.* *** *p* < 0.001; ** *p* < 0.01; * *p* < 0.05.

## Data Availability

The data used in the study are available from the corresponding author upon request.

## Supplementary Materials

The following supporting information can be downloaded at https://www.mdpi.com/article/10.3390/ejihpe15120240/s1, Table S1: Standardized factor loadings from B-ESEM model (Subsample B, N = 1020).

## Abbreviations

The following abbreviations are used in this manuscript:

EFA	Exploratory factor analysis
CFA	Confirmatory factor analysis
ESEM	Exploratory structural equation modeling
STEM	Science, technology, engineering, and mathematics
EVT	Expectancy–value theory

## Appendix A. Original Cost Items Used in This Study and Their Translations into English

	**Items (In Lithuanian)**	**Items (Translated into English)**
eff1	Po matematikos atsiskaitymų niekam daugiau neturiu jėgų.	After math tests, I do not have energy for anything else.
eff2	Tenka įdėti pernelyg daug pastangų, kad gerai mokėčiau matematiką.	I have to put too much effort to be good in math.
eff3	Turiu labai stengtis, kad suprasčiau matematiką.	I have to study very hard to understand math.
eff4	Mokantis matematikos išeikvoju pernelyg daug jėgų.	Studying math drains too much of my energy.
eff5	Man tenka įdėti daugiau pastangų norint išmokti matematiką nei kitus dalykus.	I have to put more effort into studying math than other subjects.
opp1	Norėdamas (-a) gerai mokėti matematiką, turiu riboti su draugais leidžiamą laiką.	To be good in math, I have to limit time I spend with my friends.
opp2	Dėl matematikos mokymosi turiu atsisakyti pernelyg daug kitų veiklų.	I have to give up too many other activities to study math.
opp3	Dėl matematikos mokymosi man tiesiog nelieka laiko kitoms patinkančioms veikloms.	Studying for math leaves me no time for other activities I enjoy.
opp4	Norėdamas (-a) gerai mokėti matematiką, turiu tam skirti didelę dalį savo laisvalaikio.	I have to dedicate big part of my free time to be good at math.
opp5	Norėdamas (-a) gerai mokėti matematiką, turiu atsisakyti kitų mėgstamų užsiėmimų.	I have to give up other favorite activities to be good in math.
emo1	Spręsdamas (-a) matematikos užduotis susinervinu.	I get annoyed when doing math.
emo2	Matematika mane gąsdina.	Math scares me.
emo3	Matematikos mokymasis man kelia daug įtampos.	Studying math is very stressful for me.
emo4	Matematikos atsiskaitymų bijau labiau nei kitų dalykų atsiskaitymų.	I worry more about math tests than about tests in other subjects.
emo5	Mokydamasis (-i) matematikos patiriu daug neigiamų emocijų.	I experience a lot of negative emotions when studying math.
ego1	Nusivilčiau savimi, jei gaučiau prastus matematikos pažymius.	I would be disappointed in myself if I got poor grades in math.
ego2	Man būtų gėda, jei nesugebėčiau išspręsti matematikos užduočių.	I would be embarrassed if I could not solve math problems.
ego3	Bijau, kad kitiems atrodysiu neprotingas (-a), jei nesuprasiu matematikos.	I am afraid others will think I am incapable if I do not understand math.
