# Supplementary material for: Exploring the Dimensionality of the Perceived Cost of Learning High School Mathematics
Source: Eur J Investig Health Psychol Educ. 2025 Nov 26;15(12):240. doi: 10.3390/ejihpe15120240 (PMC12731640; doi:10.3390/ejihpe15120240)
Supplement: Supplementary file 1 [file ejihpe-15-00240-s001.zip › ejihpe-3945557-supplementary.pdf]

Supplement.

Table S1. Standardized Factor Loadings from B-ESEM model (Subsample B, N = 1020).

Items	General cost factors	Effort Cost	Opportunity Cost	Emotional Cost	Ego Cost
	λ (SE)	λ (SE)	λ (SE)	λ (SE)	λ (SE)
eff2	0.732 (0.023)***	0.417 (0.043)***	0.146 (0.021)***	0.023 (0.018)	-0.057 (0.019)**
eff3	0.729 (0.024)***	0.456 (0.055)***	-0.091 (0.021)***	-0.022 (0.022)	0.009 (0.020)
eff5	0.802 (0.026)***	0.101 (0.067)	-0.027 (0.033)	-0.048 (0.041)	-0.119 (0.023)***
opp1	0.524 (0.031)***	0.172 (0.033)***	0.386 (0.036)***	-0.053 (0.033)	0.113 (0.029)***
opp2	0.702 (0.023)***	-0.022 (0.029)	0.497 (0.033)***	0.101 (0.024)***	0.018 (0.020)
opp3	0.712 (0.022)***	-0.033 (0.024)	0.527 (0.028)***	0.087 (0.020)***	-0.042 (0.022)
opp4	0.785 (0.025)***	0.047 (0.034)	0.255 (0.041)***	-0.038 (0.028)	-0.032 (0.021)
opp5	0.783 (0.023)***	-0.079 (0.029)**	0.420 (0.041)***	-0.096 (0.028)**	-0.043 (0.018)*
emo1	0.496 (0.029)***	-0.001 (0.033)	0.025 (0.025)	0.486 (0.038)***	0.062 (0.031)*
emo2	0.680 (0.025)***	-0.028 (0.027)	0.008 (0.030)	0.462 (0.034)***	0.046 (0.024)
emo3	0.713 (0.024)***	0.079 (0.029)**	0.054 (0.027)*	0.421 (0.031)***	0.081 (0.020)***
emo4	0.722 (0.026)***	-0.023 (0.056)	-0.094 (0.034)**	0.226 (0.047)***	0.004 (0.023)
emo5	0.715 (0.026)***	-0.048 (0.035)	0.020 (0.037)	0.411 (0.033)***	0.032 (0.022)
ego1	-0.013 (0.039)	0.108 (0.049)*	0.127 (0.049)*	0.134 (0.051)**	0.529 (0.029)***
ego2	0.089 (0.038)*	-0.005 (0.028)	0.004 (0.025)	0.037 (0.024)	0.802 (0.018)***
ego3	0.375 (0.036)***	0.028 (0.042)	-0.029 (0.030)	0.139 (0.035)***	0.672 (0.021)***
ego4	0.054 (0.036)	0.004 (0.030)	0.011 (0.023)	-0.014 (0.028)	0.780 (0.020)***
ego5	0.168 (0.038)***	-0.120 (0.024)***	-0.062 (0.025)*	-0.093 (0.025)***	0.890 (0.013)***

Note. ***p < 0.001; **p < 0.01; *p < 0.05; λ - Standardized factor loadings; SE – Standard errors.
